# The status of emotional labour and its influence on job burnout among village doctors during the COVID-19 pandemic in China: a cross-sectional study

**DOI:** 10.1186/s12875-023-01982-1

**Published:** 2023-01-17

**Authors:** Jia Song, Chengxin Fan, Qiusha Li, Anqi Wang, Wanchen Wang, Lifang Zhou, Haiyuan Lv, Dongping Ma, Zhongming Chen, Wenqiang Yin

**Affiliations:** 1 School of Management, Weifang Medical University, NO. 7166, Western Baotong Road, Weifang, Shandong China; 2 School of Public Health, Weifang Medical University, NO. 7166, Western Baotong Road, Weifang, Shandong China

**Keywords:** Village doctor, Emotional labour, Job burnout, COVID-19, China

## Abstract

**Background:**

Village doctors in China are not only the gatekeepers of rural residents' health but also the net bottom of the medical security system. However, emotional labour is increasingly threatening the stability of the rural primary medical system. In addition, the ongoing coronavirus disease 2019 (COVID-19) pandemic has further exposed the vulnerability of human resources in China's rural health system. This study aims to evaluate the current situation of emotional labour among village doctors and explore the impact of emotional labour on job burnout during the COVID-19 pandemic in China**.**

**Methods:**

A cross-sectional survey was conducted in December 2021 in Shandong Province. We used structured questionnaires to collect data, including sociodemographic characteristics, emotional labour, and job burnout. Data were analysed by *t test*, analysis of variance (ANOVA), Pearson correlation analysis, and hierarchical multiple linear regression.

**Results:**

A total of 1,093 village doctors from Shandong Province participated in the study. More than half of the participants were male (62.40%) and were between 41 and 50 years old (53.43%). The total mean score of emotional labour was 3.17 ± 0.67, of which the surface acting (SA) score was 2.28 ± 0.90, and the deep acting (DA) score was 3.91 ± 0.93. There were significant differences in SA according to gender and work content (*P* < 0.05) and in DA according to gender, age, education level, and work content (*P* < 0.05). Pearson correlation analysis showed that SA was positively correlated with job burnout (*P* < 0.001), and DA was negatively correlated with job burnout (*P* < 0.001). Hierarchical multiple linear regression analysis revealed that 29% of the variance in job burnout is attributable to SA (*β* = 0.530, *P* < 0.001) and DA (*β* = -0.154, *P* < 0.001).

**Conclusion:**

Village doctors in Shandong Province performed moderate levels of emotional labour during the COVID-19 pandemic. SA had a significant positive effect on job burnout, while DA had a significant negative effect on job burnout among village doctors. Administrators should enhance training on emotional labour for village doctors to play a positive role in alleviating their job burnout.

## Background

China is the world's most populous predominantly agricultural country, with a rural population of 498.35 million by the end of 2021, accounting for 35.3% of the country's total population [1]. For such a large rural population, village doctors play an indispensable role in maintaining the health of rural residents. Together with the rural three-tier health service network and the Rural Cooperative Medical System, their role has been hailed by the World Health Organization as the "three major assets" of rural health in China [2]. The predecessor of village doctors can be traced back to the "barefoot doctors", who emerged rapidly during a short period in the 1960s and reached more than 1.5 million in the 1970s [3]. Although most barefoot doctors did not have professional medical education, as a product of a specific historical period in China, they significantly alleviated the lack of medical care and medicine in rural areas under limited social conditions. In 1985, the Ministry of Health decided to discontinue using the term "barefoot doctors" and transformed this group into "village doctors" through an examination and elimination system [4]. In 2009, the Chinese government launched the New Healthcare System Reform, which is based on the principles of "ensuring the basics, strengthening the locals and constructing the mechanisms". Moreover, the reform launched the basic public health service, marking the construction of the primary health service system as one of the key tasks of China's healthcare reform [5]. Since the implementation of the New Healthcare System Reform, village doctors have been responsible for basic medical care and public health services [6]. At the same time, the number of village doctors is gradually decreasing [7], resulting in pressure on the remaining village doctors to continue practicing. The problems of low job satisfaction, low work enthusiasm, and strong burnout rates of village doctors have become increasingly prominent [8–10].

Coronavirus disease 2019 (COVID-19) is a public health emergency caused by a new infectious disease that emerged and spread rapidly worldwide in late 2019. At present, COVID-19 in China has been better controlled. China has entered a normalized phase of epidemic prevention and control, which has put forward more stringent requirements for primary care institutions. Village doctors have faced a dramatic increase in their workload for infection control, screening, cohort isolation, contact tracing, and follow-up actions for adverse symptoms after vaccination [11, 12]. Their various prevention and control efforts, such as isolating close contacts and home examinations, have caused villagers to express negative emotions and even opposition. Such negative emotions and conflicts hinder prevention work and even harm the mental health of village doctors [13]. Village doctors, who are on the front line of managing and maintaining medical services in rural areas during the COVID-19 pandemic, face not only a higher risk of infection than the general public but also risks such as psychological stress, burnout, and fatigue [14]. In addition, for one reason or another, it is more difficult to carry out prevention and control work in rural areas. For example, there is a large floating population, inconvenient transportation, and weak awareness and ability to receive information among rural residents. All these factors have brought numerous psychological and physical challenges to village doctors. Determining how to stabilize the team of village doctors and strengthen the network of epidemic prevention and control in rural areas has become an urgent issue.

Emotional labour refers to the emotional behaviour of individuals who manage their internal and external emotions to conform to organizational expectations and requirements [15], including two dimensions: surface acting (SA) and deep acting (DA). When the individual's emotional expression is inconsistent with the organizational requirements, SA means that the individual only adjusts his external emotional expression. For example, initially faced with an unknown epidemic, village doctors may hide their inner concerns, even though they may still be worried. DA means that the individual carries out a series of internal psychological activities and aligns their inner feelings with their outer expression, including their kindness in dealing with patients and their confidence in overcoming the epidemic. Both SA and DA are intended to make one's emotional expression meet organizational requirements. The difference is that there is no substantial change in the inner feelings of individuals when they perform the former, while the inner feelings of individuals are consistent with the external emotions when they perform the latter [16]. Current research has investigated emotional labour in a wide variety of occupations, including teachers [17, 18], civil servants [19], and hotel staffs [20, 21], at levels ranging from moderate to severe. More recently, researchers have shown increasing interest in emotional labour in the field of healthcare [22, 23]; however, emotional labour has not captured much attention in the primary healthcare system, which is more pronounced in developing countries [24]. Job burnout is a psychological syndrome experienced when employees operate with a heavy work load for a long time and can include emotional exhaustion (EE), depersonalization (DP), and reduced personal accomplishment (PA) [25]. Its direct consequence is the loss of motivation, enthusiasm, and efficiency for work, which indirectly makes life full of pessimism [26]. Previous studies have shown that village doctors have a high rate of job burnout, which makes them vulnerable to turnover behaviour and threatens the stability of the village doctor team [27].

The impact of emotional labour on job burnout has been widely confirmed in front-line services [28, 29], including healthcare professions [30]. However, the effect mechanism has not yet been agreed upon, and there is less research in primary care. Primary healthcare institutions are emotionally intense places, and the working environment and work characteristics make it inevitable for medical staff to take measures to perform the resultant emotional labour [31]. Previous studies have confirmed that excessive emotional labour over a long period can cause emotional exhaustion and even job burnout in individuals, which is detrimental to their physical and mental health and long-term organizational development [32]. COVID-19 is a sudden environmental disruption that places higher demands on village doctors, poses risks to their emotional health, and may even lead to burnout [33]. The data from the China Health Statistical Yearbook in 2021 showed that the number of village doctors in Shandong Province was 80,134 at the end of 2020 [34], ranking second among 31 provinces and cities in mainland China. This study took village doctors in Shandong Province as the research object, making the findings representative to some extent. This study aims to evaluate the current situation of village doctors' emotional labour and further explore its influence on their job burnout during the COVID-19 pandemic to provide valuable references for improving the current situation of village doctors' emotional labour, alleviating their job burnout and allowing them to better play their essential role in rural epidemic prevention and control.

## Methods

### Study design and sampling

This study collected data from a cross-sectional survey conducted in December 2021 in Shandong Province. Shandong Province, located in eastern China, is the third-largest economic province and the second-most populous province in China. In 2021, Shandong Province had 16 prefecture-level cities (an administrative level below the province and above the county) and a population of 101.65 million (including a rural population of 37.56 million, accounting for 36.95% of the total population). In 2020, there were 53,523 village clinics and 80,134 village doctors, with an average of 1.5 village doctors per village clinic.

In this study, stratified random sampling was used to ensure that the survey samples were representative of village doctors across Shandong Province. First, the province was divided into three regions according to its level of economic development (high, medium, and low). Second, one city in each region was randomly selected. Third, three countries were randomly selected from each city. Next, four to six townships were randomly selected from each county. Finally, twenty to thirty village doctors were randomly selected from each township. The sample size was calculated according to the following formula: *n* = [*z*^2^* *p* * (1—*p*)/*e*^2^]/[1 + (*z*^2^ * *p* * (1—*p*)/(*e*^2^ * *N*))], where *p* = 0.5, *z* = 1.96, *e* = 0.03, *N* = 80,134. The *p, z,* and *e* values are taken according to textbook recommendations [35], and the *N* value is taken from the China Health Statistics Yearbook in 2021 [34]. Ultimately, the sample size was estimated to be 1,053 participants. Meanwhile, considering that not all village doctors were on duty during the survey, we distributed 1,200 questionnaires, and 1,093 eligible questionnaires were returned, with an effective response rate of 91.1%. The inclusion criteria were as follows: village doctor who has been on the job for more than one year, was on duty when the survey was conducted, willing to participate in the questionnaire survey, and provided signed informed consent. Data were collected in the form of self-report questionnaires by village doctors. To ensure the quality of the questionnaire, the uniformly trained surveyor checked the questionnaire on the spot. If the questionnaire was found to be problematic, the respondents were invited to supplement and improve it on the spot.

### Measures instruments

#### Sociodemographic characteristics

To reduce the likelihood of sociodemographic characteristics confusing the relationships examined, we controlled for the following variables considered to be related to job burnout, which were designed based on relevant literature and expert consultation: (1) general demographic characteristics: gender [7] (male, female), age [36] (≦30, 31 ~ 40, 41 ~ 50, ≧51), marital status (married, unmarried/widowed/divorced), education level [37] (junior high school and below, high school, junior college, bachelor's degree or above); (2) work characteristics: practice qualifications (none, practice certificate of village doctors, practising assistant physician, practising physician, others), work form (full-time, medical main agricultural auxiliary, half-agriculture and half-medicine, agricultural main medical auxiliary, others), work content (only provide basic medical care, only provide basic public health services, provide basic medical care and public health services).

#### Measurement of emotional labour

We used the emotional labour scale developed by Grandey [38] and revised by Wu PJ [39]. The scale consists of 11 items divided into two dimensions: SA (five items, e.g., “You pretend to be in a positive mood at work, even when you are not internally.”) and DA (six items, e.g., “The emotions you show in your interactions with patients are consistent with the emotions you feel inside.”). All the items were rated with a five-point Likert scale, ranging from 1 (totally disagree) to 5 (totally agree). Higher scores indicated more emotional labour for village doctors. Our study reported a coefficient alpha of 0.78 for the total score, 0.85 for Subscale 1: SA, and 0.88 for Subscale 2: DA. These values are above 0.7, which is within the acceptable range, indicating that the scale has good reliability [40].

#### Measurement of job burnout

We used the Chinese Maslach Burnout Inventory of Li et al. [41], which was revised based on the Maslach Burnout Inventory-General Survey (MBI-GS) questionnaire [42]. This questionnaire has 15 items with a five-point Likert scale ranging from 1 (totally disagree) to 5 (totally agree). It consists of three subscales, namely, EE (five items, e.g., “Work makes you feel physically and mentally exhausted.”), DP (four items, e.g., “Since you took on this work, you have become increasingly disinterested in it.”), and PA (six items, e.g., “You can effectively solve the problems arising in your work.”). PA is a negative dimension, so it is reverse scored. Higher scores of EE and DP and lower scores of PA indicate higher risks of job burnout. In our study, Cronbach's alpha coefficients for the total score and its three subscales—EE, DP, and PA—were 0.88, 0.93, 0.82, and 0.87, respectively, indicating that the scale has good reliability.

### Statistical analysis

The statistical analyses were performed with SPSS version 26 (IBM Corp, Armonk, NY, USA). Count data were described by composition ratio, and measurement data were described by the mean and standard deviation (*M* ± *SD*). A *t test* and ANOVA were used to assess the differences in emotional labour and job burnout scores of village doctors in terms of sociodemographic characteristics. Pearson correlation analysis was used to measure the correlation between emotional labour and job burnout. Hierarchical multiple linear regression analysis was performed with the total score of job burnout as the dependent variable and dimensions of emotional labour as the independent variable. (α into = 0.05, α out = 0.10). The first model (Model 1) included sociodemographic variables (gender, work form, and work content) with significant differences in job burnout, which were set as controlled variables for the final inclusion in the study. Among them, work form and work content were categorical variables without a linear trend, and dummy variables were set for these. The second model (Model 2) added dimensions of emotional labour (SA and DA). This enables an examination of the variance in job burnout that emotional labour may explain in addition to the variance explained by control variables. The significance level of all tests was set at *P* < 0.05 (two-tailed).

## Results

### Sociodemographic characteristics

Among the 1,093 village doctors, 62.40% were male, and more than half (53.43%) were between 41 and 50 years old. The respondents were predominantly (95.97%) married. The participants' education level ranged from junior high school and below (1.46%) to bachelor's degree or above (7.50%). Among them, 54.62% were high school graduates. In terms of the practice qualification, respondents who had village doctors' practice certificates accounted for 58.46%, which was higher than those others. Furthermore, 43.37% of respondents were mainly full-time. Most of the respondents (87.74%) provided basic medical care and public health services.

### Description of emotional labour and job burnout

In Table 1, the total mean score of emotional labour was 3.17 ± 0.67, in which the SA score was 2.28 ± 0.90, and the DA score was 3.91 ± 0.93. The total mean score of job burnout was 2.36 ± 0.59, and the mean scores of EE, DP, and PA were 3.05 ± 0.94, 2.12 ± 0.85, and 1.95 ± 0.61, respectively.

**Table 1:** Emotional labour and job burnout scores (*n* = 1,093)

Items	*M* ± *SD*
The total mean score of emotional labour	3.17 ± 0.67
SA	2.28 ± 0.90
DA	3.91 ± 0.93
The total mean score of job burnout	2.36 ± 0.59
EE	3.05 ± 0.94
DP	2.12 ± 0.85
PA	1.95 ± 0.61

*SA* Surface acting, *DA* Deep acting, *EE* Emotional exhaustion, *DP* Depersonalization, *PA* Reduced personal accomplishment

### Univariate analysis of emotional labour and job burnout

Given that the core independent variables of this study are the two dimensions of emotional labour, namely, SA and DA, these two are reported separately. The results of the univariate analysis of SA showed that the differences in the total SA scores of village doctors compared by gender and work content were statistically significant (*P* < 0.05). Village doctors who were male, provided basic medical care and public health services at a higher level of SA. The results of the univariate analysis of DA showed that the differences in the total DA scores of village doctors comparing gender, age, education level, and work content were statistically significant (*P* < 0.05). Village doctors who were female, aged 41–50, with a bachelor's degree or above, only provide basic medical care at a higher level of DA (Table 2).

**Table 2:** Sociodemographic characteristics of participants (*n* = 1093)

Sociodemographic Variable	Composition ratio (%)	Surface acting			Deep acting			Job burnout		
		**Score**	***t/F***	***P value***	**Score**	***t/F***	***P value***	**Score**	***t/F***	***P value***
**Gender**			1.990^a^	0.047^*^		-2.689^a^	0.007^**^		2.217^a^	0.027^*^
Male	682 (62.40)	2.33 ± 0.89			3.85 ± 0.95			2.39 ± 0.59		
Female	411 (37.60)	2.21 ± 0.90			4.00 ± 0.89			2.31 ± 0.59		
**Age (years)**			1.552^b^	0.199		2.817^b^	0.038^*^		1.462^b^	0.223
≦30	17 (1.56)	2.11 ± 0.76			3.62 ± 1.28			2.15 ± 0.55		
31 ~ 40	211 (19.30)	2.29 ± 0.86			3.83 ± 0.94			2.39 ± 0.59		
41 ~ 50	584 (53.43)	2.33 ± 0.91			3.98 ± 0.90			2.37 ± 0.58		
≧51	281 (25.71)	2.20 ± 0.90			3.83 ± 0.97			2.32 ± 0.59		
**Marital status**			-0.627^a^	0.531		0.406^a^	0.685		2.206^a^	0.138
Married	1049 (95.97)	2.29 ± 0.90			3.90 ± 0.93			2.37 ± 0.58		
Unmarried/widowed/divorced	44 (4.03)	2.20 ± 0.94			3.96 ± 1.09			2.23 ± 0.68		
**Education level**			1.092^b^	0.351		3.886^b^	0.009^**^		0.404^b^	0.750
Junior high school and below	16 (1.46)	1.95 ± 0.74			3.22 ± 1.30			2.27 ± 0.52		
High school	597 (54.62)	2.29 ± 0.92			3.87 ± 0.93			2.37 ± 0.58		
Junior college	398 (36.41)	2.27 ± 0.87			3.97 ± 0.90			2.34 ± 0.60		
Bachelor's degree or above	82 (7.50)	2.38 ± 0.88			3.98 ± 0.98			2.38 ± 0.59		
**Practice qualifications**			0.626^b^	0.644		0.436^b^	0.783		0.396^b^	0.812
None	14 (1.28)	2.20 ± 0.94			3.92 ± 1.11			2.28 ± 0.47		
Practise certificate of village doctors	639 (58.46)	2.25 ± 0.91			3.89 ± 0.94			2.35 ± 0.58		
Practising assistant physician	296 (27.08)	2.34 ± 0.87			3.95 ± 0.91			2.39 ± 0.58		
Practising physician	135 (12.35)	2.31 ± 0.91			3.93 ± 0.93			2.39 ± 0.65		
Others	9 (0.82)	2.38 ± 1.16			3.65 ± 0.83			2.35 ± 0.82		
**Work form**			1.530^b^	0.191		0.474^b^	0.755		4.316^b^	0.002**
Full-time	474 (43.37)	2.22 ± 0.92			3.93 ± 0.95			2.29 ± 0.61		
Medical main agricultural auxiliary	149 (13.63)	2.26 ± 0.89			3.95 ± 0.89			2.34 ± 0.55		
Half-agriculture and half-medicine	423 (38.70)	2.35 ± 0.89			3.86 ± 0.95			2.44 ± 0.57		
Agricultural main medical auxiliary	41 (3.75)	2.44 ± 0.78			3.89 ± 0.77			2.51 ± 0.55		
Others	6 (0.55)	2.27 ± 0.78			3.94 ± 0.75			2.38 ± 0.77		
**Work content**			5.409^b^	0.005^**^		8.686^b^	< 0.001^***^		8.586^b^	< 0.001^***^
Only provide basic medical care	75 (6.86)	1.98 ± 0.95			4.08 ± 0.86			2.09 ± 0.59		
Only provide basic public health services	59 (5.40)	2.16 ± 0.92			3.45 ± 1.15			2.36 ± 0.67		
Provide basic medical care and public health services	959 (87.74)	2.31 ± 0.89			3.92 ± 0.92			2.38 ± 0.58		

^*^ *P* < 0.05

^*^^*^ *P* < 0.01

^**^^*^ *P* < 0.001

^a^ results of independent sample *t test*

^b^ results of one-way analysis of variance

The results of the univariate analysis of job burnout showed that the differences in the total job burnout scores of village doctors comparing gender, work form, and work content were statistically significant (*P* < 0.05). Job burnout levels were higher among village doctors who were male, had agricultural main medical auxiliary, and provided basic medical care and public health services (Table 2).

### Correlation analysis

In Table 3, we used Pearson correlation analysis to explore the correlation between the dimensions of emotional labour and job burnout. The results showed that there was a positive correlation between SA and job burnout (*r* = 0.533, *P* < 0.001) and a negative correlation between DA and job burnout (*r* = -0.132, *P* < 0.001).

**Table 3:** Correlation analysis of emotional labour and job burnout among village doctors (*n* = 1093)

Factors	Job burnout	
	***r*-value**	***P value***
SA	0.533	< 0.001
DA	-0.132	< 0.001

*SA* Surface acting, *DA* Deep acting

### Hierarchical multiple linear regression analysis

Table 4 reports the results of hierarchical regression linear analysis, which was performed to explore the relationship between emotional labour and job burnout. It should be mentioned that the range of tolerance was 0.56–0.99 and that of the variation inflation factor (VIF) was 1.01–1.80, indicating no risk of multicollinearity between independent variables. Furthermore, the Durbin-Watson statistic for testing the independence of the residuals was 1.75, confirming no autocorrelation among the error terms.

**Table 4:** Hierarchical multiple linear regression analysis of village doctors' emotional labour on job burnout (*n* = 1093)

Variables	Model 1			Model 2		
	***β***	***t***	***VIF***	***β***	***t***	***VIF***
**Controlled variable**						
Gender	-0.040	-1.312	1.058	-0.004	-0.169	1.067
Work form						
Work form_1	0.010	0.320	1.169	0.015	0.539	1.170
Work form_2	0.094	2.810^**^	1.244	0.068	2.411^*^	1.246
Work form_3	0.057	1.846	1.076	0.040	1.550	1.077
Work form_4	0.024	0.782	1.022	0.016	0.628	1.022
Work content						
Work content_1	0.090	2.287^*^	1.728	0.047	1.401	1.754
Work content_2	0.142	3.543^***^	1.784	0.075	2.235^*^	1.799
**Independent variable**						
SA				0.530	20.925^***^	1.019
DA				-0.154	-6.061^***^	1.028
*R*^*2*^	0.029			0.319		
*Adjusted R*^*2*^	0.022			0.313		
Δ*R*^*2*^	0.029			0.290		
*F*	4.555^***^			56.310^***^		

Gender, male versus female; Work form: Work form_1, medical main agricultural auxiliary versus full-time; Work form_2, half-agriculture and half-medicine versus full-time; Work form_3, agricultural main medical auxiliary versus full-time; Work form_4, others versus full-time. Work content: Work content_1, only provide basic public health services versus only provide basic medical care; Work content_2, provide basic medical care and public health services versus only provide medical care. SA, original values were entered; DA, original values were entered

^*^ *P* < 0.05

^**^ *P* < 0.01

^***^ *P* < 0.001

The results showed that SA and DA were predictive of job burnout in village doctors (*F* = 56.310, *P* < 0.001), with an increase in *R*^*2*^ from 0.029 to 0.319 and a net explained variance of 29%. SA was positively associated with job burnout (*β* = 0.530, *P* < 0.001), whereas DA was negatively associated with job burnout (*β* = -0.154, *P* < 0.001).

## Discussion

The results of this study showed that the emotional labour mean score of village doctors was 3.17 ± 0.67, which was a moderate level and higher than the findings by Qian et al. [43] on front-line medical personnel in tertiary hospitals. This discrepancy could be attributed to village doctors having geo-blood relations with villagers, and the lack of medical knowledge among rural residents also makes the doctor‒patient relationship between village doctors and villagers more complicated [44]. Therefore, village doctors need to constantly adjust their emotional behaviour according to the actual situation and display appropriate emotional behaviour to facilitate smooth communication with patients. In addition, village doctors in this study were more inclined to use DA, with a score of 3.91 ± 0.93, much higher than a survey of Jordanian doctors [45]. One possible reason is that village doctors are responsible for family doctor contract services and need to regularly follow up with chronic patients in their districts, so they are closer to the residents and prefer to change both inner psychological feelings and outer behavioural performance when performing emotional labour. Another reason might be that Shandong Province is the birthplace of traditional Confucian culture, where village doctors are deeply influenced by Confucianism and tend to absorb negative emotions internally when dealing with exhausting work. Furthermore, our study showed that SA and DA are highly correlated, which confirms that SA and DA are not an either/or relationship but can be adopted by village doctors simultaneously. This is consistent with the results of previous studies [46, 47]. That is, village doctors who face negative emotions at work may first hide their negative emotions and then actively try to align their internal and external emotional performance through internal emotional regulation [48].

Sociodemographic factors; including gender, age, education level, and work content; were found to be related to emotional labour among village doctors. In our study, male village doctors were found to use SA more frequently, while female village doctors used DA more frequently. This difference may be related to women's greater flexibility in emotional regulation [49]. In terms of age, village doctors aged 41–50 years old experienced a higher level of DA. This finding could be explained by this age group having a greater understanding of family influence in traditional Chinese culture and more experience in creating and maintaining a balance between personal life and professional life. This would also suggest that village doctors who have more experience may be more likely to regulate their emotional displays in effective ways [50]. Moreover, village doctors with a bachelor's degree or above have higher DA. One plausible explanation could be that mechanical, simple, and repetitive work inevitably makes a significant deviation between the professional role of village doctors and their self-worth, so they have more negative emotions and have to adjust their emotions constantly. On the other hand, a higher level of education usually implies that their ability to receive training related to emotional labour is higher and that they are more likely to apply it in practice. It is worth mentioning that village doctors who provide basic medical care and public health services have higher levels of both SA and DA. During home quarantine, village doctors, who are responsible for basic medical care and public health services, fully utilize their medical service functions, such as delivering medicine. On the one hand, this effectively ensures that villagers receive medical services on time. On the other hand, it increases the workload of village doctors, leading them to generate plenty of emotions. This group should receive particular attention from managers to guide them to transform SA into DA.

As our results indicated, after controlling for sociodemographic variables, emotional labour independently explained 29% of job burnout among village doctors, indicating that emotional labour had a significant impact on job burnout among village doctors. Further analysis revealed that the absolute value of the regression coefficient of SA was much larger than that of DA, indicating that village doctors had the greatest impact on their job burnout level when they used SA. At the same time, SA has a positive effect on job burnout, whereas DA has a negative effect on job burnout. This is in line with previous studies [51, 52]. In other words, the more village doctors use SA, the higher their job burnout level is, while the more they adopt DA, the lower their job burnout level is. According to resource conservation theory, village doctors who adopt SA strategies will not only create internal conflicts due to the inconsistency between their inner feelings and emotional expressions but also consume more resources to continuously monitor their emotional expressions [53], while DA focuses on adjusting their inner experience from the perspective of cognitive evaluation. This mental adjustment will cause more emotional activation, which is a process of resource acquisition [54]. Furthermore, when village doctors adopt DA from the heart, their internal emotions and external emotional behaviours remain coordinated and consistent. Therefore, it is possible to dedicate sincere feelings to the patients and obtain positive emotional feedback from them, which plays a positive role in alleviating job burnout among village doctors, to a certain extent [55]. These results all showed that village doctors should be motivated to adopt DA instead of SA as much as possible to alleviate their job burnout.

Given the current situation of village doctors in China, which is characterized by a low educational background and old age [56], they are unfamiliar with the concept of "emotional labour" and have not considered using emotional labour to perfect their daily work. Consequently, it is particularly significant to improve the current situation of the emotional labour of village doctors from the organizational management level. First, administrators should make efforts to create a relaxed, harmonious, and positive cultural environment to encourage village doctors to use more DA than SA. For instance, village doctors who have contributed to the prevention and control of the epidemic should be awarded the title of "Most Beautiful Village doctor". Second, administrators should pay more attention to training village doctors on emotional labour, teaching them to strike a balance between emotional involvement and emotional detachment, avoiding excessive emotional involvement, thus protecting themselves from suffering emotionally and stressful situations and fostering their physical and mental health. Furthermore, improved material working conditions may influence psychological responses, supporting village doctors in adopting more positive emotional labour strategies. For example, research interviews revealed that several cities in Shandong Province have established a level of guaranteed revenue and a basic pension insurance system for village doctors. Improving the remuneration of village doctors increases their emotional harmony, reduces burnout, and stabilizes the village doctor workforce.

This study introduced emotional labour to primary healthcare and focused on the job burnout of front-line medical personnel in rural China during the COVID-19 pandemic, which is meaningful for epidemic prevention and control. Even though it followed a stringent protocol in study design, several limitations must be acknowledged. First, our study was a cross-sectional investigation and cannot determine the causal relationship between the results and the variables. Longitudinal research is needed in the future. Second, we only investigated village doctors on duty, while absent village doctors may have been more negatively affected by emotional labour, thus biasing our findings. In future research, the data can be supplemented by distributing questionnaires through the internet. Third, this study focused only on the associations of emotional labour with job burnout. Other factors, such as job satisfaction and work engagement, which are also important factors that influence job burnout, were not included. Further studies that take these factors into account will need to be undertaken.

## Conclusions

Our study found that village doctors in Shandong Province performed moderate levels of emotional labour during the COVID-19 pandemic. SA differed depending on gender and work content, while DA differed depending on gender, age, education level, and work content. Moreover, as we showed, village doctors' job burnout was influenced by their emotional labour; SA had a negative effect on job burnout among village doctors, while DA had a positive effect on it. It is suggested that administrators should take adequate measures to motivate village doctors to consciously adopt DA to reduce the level of job burnout and improve the stability of the village doctors' workforce.

## Data Availability

The datasets generated and analyzed during the current study are not publicly available due to data analysis has not been completed but are available from the corresponding author on reasonable request.

## Abbreviations

COVID-19: Coronavirus disease 2019
ANOVA: Analysis of variance
SA: Surface acting
DA: Deep acting
EE: Emotional exhaustion
DP: Depersonalization
PA: Reduced personal accomplishment
VIF: Variation inflation factor
